# Safety Assessment of 2D MXenes: In Vitro and In Vivo

**DOI:** 10.3390/nano12050828

**Published:** 2022-03-01

**Authors:** Jialong Wu, Yanyan Yu, Gaoxing Su

**Affiliations:** 1 Key Laboratory for Water Quality and Conservation of the Pearl River Delta, Ministry of Education, Institute of Environmental Research at Greater Bay, Guangzhou University, Guangzhou 510006, China; 2111904062@e.gzhu.edu.cn; 2 School of Pharmacy, Nantong University, Nantong 226001, China

**Keywords:** MXenes, 2D materials, toxicology, safety assessments, in vitro, in vivo

## Abstract

MXenes, representing a new class of two-dimensional nanomaterial, have attracted intense interest in a variety of fields as supercapacitors, catalysts, and sensors, and in biomedicine. The assessment of the safety of MXenes and related materials in biological systems is thus an issue that requires significant attention. In this review, the toxic effects of MXenes and their derivatives are summarized through the discussion of current research into their behaviors in mammalian cells, animals and plants. Numerous studies have shown that MXenes have generally low cytotoxicity and good biocompatibility. However, a few studies have indicated that MXenes are toxic to stem cells and embryos. These in vitro and in vivo toxic effects are strongly associated with the dose of material, the cell type, the mode of exposure, and the specific type of MXene. In addition, surface modifications alter the toxic effects of MXenes. The stability of MXenes must be considered during toxicity evaluation, as degradation can lead to potentially toxic byproducts. Although research concerning the toxicity of MXenes is limited, this review provides an overview of the current understanding of interactions of MXenes with biological systems and suggests future research directions.

## 1. Introduction

Due to the remarkable properties of graphene, two-dimensional (2D) materials have attracted extensive scientific and engineering interest. In 2011, a novel class of inorganic 2D materials named MXenes was discovered by scientists at Drexel University [1]. MXenes are represented by the general chemical formula M*_n_*_+1_X*_n_*T*_x_*, in which M represents an early transition metal (e.g., Ti, V, Sc, Nb, Zr, Mo, Hf, or Ta), X is C and/or N, *n* = 1 to 6, and T*_x_* stands for the surface termination groups (typically, –O, –OH, –F, –Cl, –Br, or –S). To date, more than 30 stoichiometric MXenes have been experimentally produced, and over 70 MXenes have been theoretically predicted [2]. Among them, Ti_3_C_2_T*_x_* is the most widely studied.

MXenes are fabricated by etching layers of A-elements including Al, Si, Ga, and Ge, containing the parent transition metal carbides and/or nitrides in the so-called MAX phase. The A layers are selectively etched without destroying the M−X bonds because of the stronger chemical activity of M−A bonds relative to M−X bonds. As the resulting 2D M-X materials have graphene-like morphologies, they have been termed “MXenes”.

MXenes exhibit favorable properties for use in electronics, mechanics, magnetism, and optics because the layers are only a few atoms thick [3]. The use of MXenes in multiple fields, especially in biomedical applications, is facilitated by abundant polar surface functional groups that make MXenes hydrophilic. In addition, the preparation of MXenes is relatively easy and can be readily scaled up. These properties make MXenes valuable to a variety of fields, including supercapacitors [4], catalysis [5], sensors [6], electrochemical energy storage [2], optoelectronics [7], environmental remediation [8,9], electromagnetic interference shielding [10] and biomedicine [11,12].

With the rapid growth in the production and application of MXenes, the release of these materials into the environment is inevitable, and potential toxicities of MXenes should be carefully evaluated. Interactions with physiological systems is especially important due to the increasing interest in biomedical applications of MXenes, including in anticancer therapy [13], antibacterial therapy [14], biosensing [15,16], bioimaging [17], radioprotection [18], combined therapy and theranostics [11,19]. Before these materials are approved for clinical use, the basic interactions with physiological systems should be understood, and evaluations of biocompatibility are essential. The assessment of the safety of MXenes is important both regarding the use of MXenes in nanomedicine and regarding implications for the synthesis and widespread use of MXenes for the environment.

Once nanomaterials come in contact with cells, they could be internalized into cells or adhered to cell membranes. Intracellular homeostasis may then be disrupted due to the physical presence of the nanomaterial and its interactions with organelles, cell membranes, proteins, lipids, and other cellular structures. This disruption may lead to apoptosis [20], autophagy [21], neurosis [20], immunostimulation [22], and other deleterious effects. Two-dimensional materials with ultra-thin nanostructures are likely to enter into multiple cell types [23], and cytotoxicity has been observed experimentally [24]. In vivo studies have demonstrated that 2D materials can accumulate in organs including liver, spleen, kidneys and lungs. Numerous in vivo and in vitro investigations into mechanisms and consequences exposure to 2D ultrathin nanostructures have been reported [24,25]. However, since MXenes are relatively new 2D materials, a limited number of studies regarding the toxicity of this material have been carried out. Some of these studies focus on the biomedical applications of MXenes, and evaluations of toxic effects are merely tangential aspects of the work. Generally, MXenes have been reported as having no obvious toxicity in these cell-based or short-term experiments. Thus far, deep investigations about physiological influences and basic interactions between MXenes and physiological environments have been rare, but they are in high demand.

In this review, we first introduce the synthesis of MXenes, paying particular attention to important hazards related to the synthetic procedures. Next, recent studies regarding interactions between MXenes and various classes of mammalian cells and tissues are summarized, while noting that the toxicology and mechanisms of toxicity vary depending on cell type and route and extent of exposure. The toxicological profiles also depend on the physicochemical properties of the MXenes, including stability and surface chemistry. Finally, we conclude this review with future perspectives for the assessment of the safety production and application of MXenes.

## 2. Synthesis of MXenes and Safety Concerns

**Figure 1:**
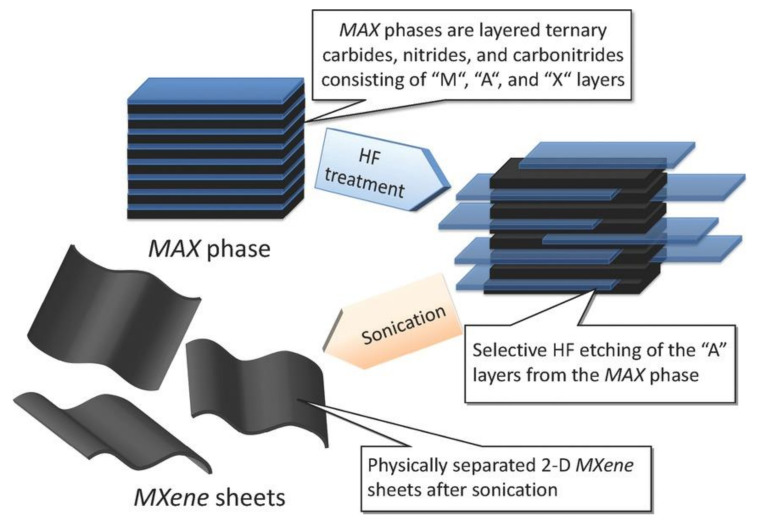
Synthesis of MXenes from the corresponding MAX phases. Reproduced with permission from [27]. Copyright American Chemical Society, 2012.

Among the multiple bottom-up and top-down approaches to MXene synthesis, etching of A-element layers from the MAX phase is the most popular strategy (Figure 1) [1,26,27]. In this process, the MAX phase powder is stirred in aqueous hydrofluoric acid (HF), and after an appropriate amount of time, the solid is separated from the supernatant by centrifugation or filtration. After washing, the dense MAX particles are converted to a loosely packed accordion-like structure, which is referred to as multilayer MXenes. Subsequent gentle sonication results in the exfoliation of MXene nanosheets, and 10 or fewer layers of terminated MXenes, with thicknesses of approximately 10 nm, are ultimately obtained.

**Figure 2:**
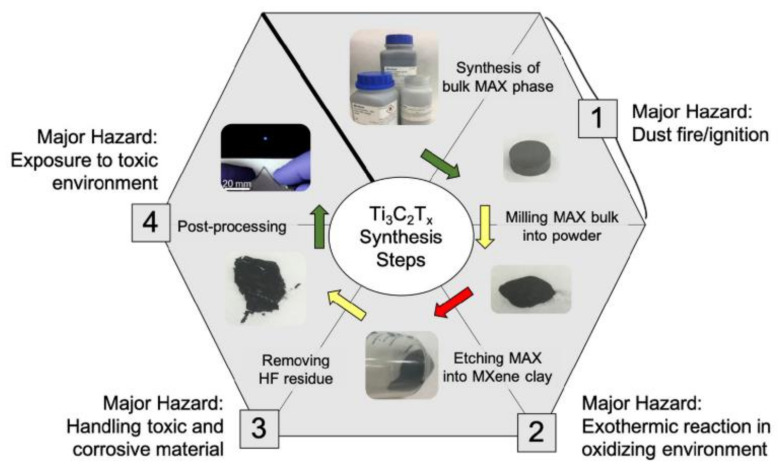
Safety concerns in the synthesis of Ti_3_C_2_T*_x_* from raw materials. Reproduced with permission from [28]. Copyright American Chemical Society, 2019.

As the production of MXene materials is scaled up to commercial levels, it is important to analyze the safety of the synthetic procedure. Dust ignition, runaway reactions, and toxic chemical exposure are the most important potentially hazardous factors in MXene synthesis (Figure 2) [28]. Firstly, in MAX phase synthesis, hazards associated with dust production are present in both reactant handling and postsynthesis processing procedures. Using alternative milling methods may increase safety.

Next, a significant hazard exists in the process of etching the Al layer in the MAX phase. If the Ti_3_AlC_2_ MAX phase is added too quickly, corrosive materials can leave containment, and toxic fumes can be produced. In particular, the most widely used etching methods employ HF, which is extremely toxic and containment of HF represents a significant challenge to the industry. When using HF, HF-specific personal protective equipment should be worn to minimize HF exposure [29]. Additionally, if the Ti_3_C_2_T*_x_* clay is not properly washed, HF-related hazards persist throughout the procedure, even into postprocessing, and this durability adds to the toxicity found through in vitro and in vivo studies.

Due to the hazardous nature of HF, researchers have explored an alternative milder etching processes, including electrochemistry [30], alkali treatment [31], hydrothermal methods [32] and the use of Lewis acidic molten salts [33]. In addition to using sonication for exfoliation, organic solvents including dimethyl sulfoxide, *n*-butylamine, tetrabutylammonium hydroxide and choline hydroxide have been investigated for the generation of MXenes [3,34]. Furthermore, multilayer MXenes can be immersed in such solvents to swell, which with further sonication would result in the formation of colloidal particles. After centrifugation, homogeneous MXene nanosheets were prepared.

## 3. In Vitro Toxic Effects of MXenes

Toxicity concerns are not only associated with the production of MXenes, in addition, the use of MXenes, especially in medical related application, may be associated with safety issues. Cell-based assays are usually initially employed to evaluate the toxic effects of engineered nanomaterials in vitro (Table 1). As Ti_3_C_2_T*_x_* nanosheets are the most widely applied MXenes, the cytotoxicity of this material was the first to be evaluated. Zhang et al. revealed that there was no obvious acute cytotoxicity to human umbilical vein endothelial cells (HUVECs) after treatment with two different concentrations of Ti_3_C_2_T*_x_* nanosheets (100 and 500 μg mL^−1^) [35]. While scanning electron microscopy (SEM) images of MXene-treated cells showed that Ti_3_C_2_T*_x_* nanosheets contacted cell membranes and formed spherical structures, the ratios of living, apoptotic, and necrotic cells after treatment were similar to those of the control group. However, at a high dose, a significant metabolic shift was observed in HUVECs, which was reflected as the enhancement of glycolysis, fatty acid biosynthesis, and lipid accumulation. On the other hand, in experiments using HUVECs as an in vitro model, Gu et al. found that Ti_3_C_2_ quantum dots (QDs) exhibited higher cytotoxic effects than did Nb_2_C QDs [36]. After treatment with 25 μg mL^−1^ of Ti_3_C_2_ QDs, the cellular viability was greater than 70%, but higher concentrations (50 and 100 μg mL^−1^) of Ti_3_C_2_ QDs led to significantly lower cellular viability as compared with Nb_2_C QDs. For Nb_2_C QDs, there was no significant cytotoxicity after being treated with these three concentrations. Due to the different cytotoxicities of MXene QDs relative to other forms of MXene, it is likely that the MXene structure has an important impact on its toxicity.

Treating human mesenchymal stem cells (hMSCs) with a high dose of Ti_3_C_2_T*_x_* nanosheets (>50 μg mL^−1^) led to obvious cytotoxicity, as shown in [37]. At a dose of 100 μg mL^−1^, hMSCs displayed a significant decrease of cell proliferation early in this study. As for cells treated with 50 μg mL^−1^ of MXenes, the first decrease of cell proliferation was noted after 5 days of incubation.

In a similar finding from another lab [38], Ti_3_C_2_T*_x_* nanosheets were shown to induce dose-dependent cytotoxicity to primary neural stem cells (NSCs) and NSC-derived differentiated cells. At a dose of 12.5 μg mL^−1^, Ti_3_C_2_T*_x_* nanosheets exhibited no observable adverse effects on NSCs or NSCs-derived differentiated cells. However, at 25 μg mL^−1^, Ti_3_C_2_T*_x_* nanosheets caused significant cytotoxicity and were internalized into the NSCs upon compromising of the cell membrane. Subsequent biological assays revealed that the 25 μg mL^−1^ dose significantly increased the rate of apoptosis of the NSCs. Furthermore, 198 differently expressed genes (DEGs), which were mainly associated with the extracellular regions, were identified. The characteristics of these DEGs suggested that the decrease of viability of NSCs might due to the disruption of the cell membrane upon internalization of nanosheets at high doses.

In addition to being dependent on the dose, the in vitro toxic effects of MXenes are also dependent on the target cell types. Jastrzebska et al. investigated the cytotoxicity in two normal (MRC-5 and HaCaT) and two cancerous (A549 and A375) cell lines [39]. For Ti_3_C_2_T*_x_* nanosheets, the toxic effects were higher against the cancerous cells in comparison to normal cells. Scheibe et al. also found that the short-term cytotoxicities of different Ti_3_C_2_T*_x_* MXene structures and their precursors (TiC, Ti_2_AlC, and Ti_3_AlC_2_) were higher when they were applied to cancer-derived HeLa cells than when they were applied to normal human fibroblasts [40]. The higher ROS generation and stronger adhesion to cell membranes brought about higher cytotoxicity to cancer cells. In addition, for HeLa cells, MXene structures showed higher cell viability in comparison to MAX phases.

Previous studies have demonstrated that oxidative stress was the most probable cause of the cytotoxicity of 2D materials. However, images from confocal laser scanning microscopy demonstrated higher generation of reactive oxygen species (ROS) upon treatment with MXenes in comparison to their precursor MAX phases. As MXenes tend to be more toxic, this finding indicated that cytotoxicity pathways are not strongly dependent on the ROS production, but rather on direct physicochemical interactions of larger particles with the cell membranes. These mechanisms were further confirmed through the evaluation of the cytotoxicity of the Ti_2_NT*_x_* MXene [41]. In this experiment, Ti_2_NT*_x_* nanosheets also showed higher toxicity towards cancerous cell lines in comparison to normal ones, but intracellular ROS production only increased in A375 cells. Meanwhile, in each normal cell line, and in MCF-7 cells, their ROS-specific fluorescence remained constant over time and relative to controls. Therefore, the mechanism of cytotoxicity of Ti_2_NT*_x_* nanosheets was not solely dependent on the generation of ROS, but instead, internalization of the 2D sheets and cell membrane disruption were also the mechanisms leading to the toxic effects of MXenes.

Notably, the cytotoxicity of MXenes has been applied in a therapeutic way. Due to the differential toxic effects of MXenes towards normal cells, and because MXenes have been shown to have photothermal effects, MXenes have been developed as cancer phototherapeutic agents [13]. Two cancer cell lines, breast 4T1 cancer cells and glioma U87 cancer cells, were treated with polyvinyl pyrrolidone (PVP)-coated Nb_2_CT*_x_* nanosheets at various concentrations from 0 to 200 μg mL^−1^. After a 24 h incubation, Nb_2_CT*_x_* nanosheets showed negligible effects on the survival of both 4T1 and U87 cells, even the highest dose. However, after incubation with 40 μg mL^−1^ Nb_2_CT*_x_* nanosheets for 4 h followed by infrared laser irradiation (λ = 808 or 1064 nm), the cancer cells exhibited significant growth inhibition. The same lab also performed similar photothermal therapy using soybean phospholipid-modified Ti_3_C_2_T*_x_* nanosheets. These nanomaterials showed negligible effect on the viability of 4T1 cells, even at a dose of 400 μg mL^−1^ [42]. When Ti_3_C_2_T*_x_* nanosheets and near infrared laser irradiation were applied the majority of 4T1 cells were killed, suggesting that the increased intracellular temperature promoted cancer cell ablation.

**Figure 3:**
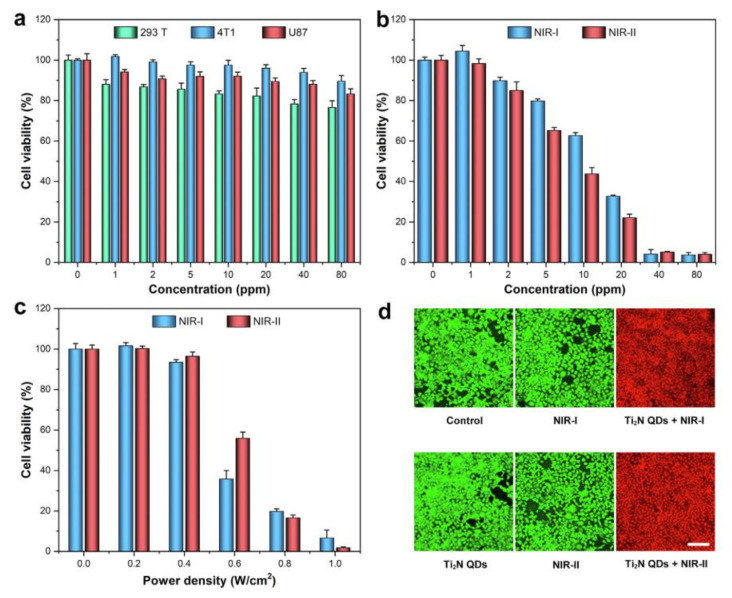
(**a**) Cell viability of 293T, 4T1, and U87 cells after 24 h incubation with different concentrations of Ti_2_N QDs. (**b**) Cell viability of 4 T1 cells co-cultured with different concentrations of Ti_2_N QDs for 4 h after 5 min irradiation with 808 or 1064 nm lasers. (**c**) Cell viability of 4T1 cells after incubation with Ti_2_N QDs (40 μg mL^−1^) irradiated with 808 or 1064 nm laser at different power densities for 5 min. (**d**) Confocal fluorescence images of calcein-AM (green, live cells) and PI (red, dead cells) co-stained cells after the treatment (scale bars: 100 μM). Reproduced with permission from [43]. Copyright Elsevier, 2020.

In addition, Ti_2_N QDs have also been explored as photothermal therapeutic agents [43]. At a dose of 80 μg mL^−1^ Ti_2_N QDs exhibited no cytotoxicity to all tested cells, including 293T human embryonic kidney cells, 4T1 murine mammary carcinoma cells, and U87 human malignant glioma cells (Figure 3). However, supplementing this treatment with near infrared laser irradiation for 5 min led to almost all of the cancer cells being killed. These studies suggested tremendous potential for the use of MXenes in cancer treatment.

Furthermore, 3D cell culture systems have also been used to evaluate the cytotoxicity of Ti_2_CT*_x_* MXene in vitro [44]. Using microscopy, it was observed that MXenes possessed a strong binding capacity to the cellular membranes of HeLa cells due to the electrostatic interactions between the negatively charged MXenes and positively charged proteins on the cell surface. Accordingly, cell viability decreased from 98% to 43% as the dose of Ti_2_CT*_x_* MXenes increased from 50 to 300 μg mL^−1^, indicating that the cytotoxicity was dose dependent. In the 3D cell culture system, the cytotoxicity of MXenes was evaluated by dispersing the Ti_2_CT*_x_* MXenes at doses of 50 μg mL^−1^ and 500 μg mL^−1^, among HeLa cells encapsulated in alginate microbeads. Using fluorescence microscopy, dead cells were observed in the area where the agglomerates of Ti_2_CT*_x_* MXenes directly bound to the surface of the 3D microtissues. The direct adhesion of Ti_2_CT*_x_* MXenes to the cell membranes of microtissues limited the growth of microtissues and cell division, resulting in lower cell viability. Importantly, oxidative stress assays showed that there was no significant difference in ROS level between control cells and Ti_2_CT*_x_* MXene-treated cells, meaning that the cytotoxicity pathways of Ti_2_CT*_x_* MXene were not associated with oxidative stress, but instead were influenced by the direct physicochemical interactions of Ti_2_CT*_x_* MXene with the cell membrane.

As in vitro cytotoxicity evaluations are time-consuming and expensive, computational machine learning methods have recently been applied to predict the interactions between MXenes and cells. Marchwiany et al. constructed the predictive model of 2D MXenes and identified that the most relevant highly surface-specific features that might be responsible for the cytotoxic behavior of MXenes [45]. The machine learning models predicted that two types of 2D MXenes could exhibit cytotoxic properties with high probabilities, while other MXenes studied were predicted to be non-toxic. In the non-toxic MXenes, there was no presence of M*_x_*O*_y_* on the surface, suggesting that the presence of M*_x_*O*_y_* was the key toxicity-generating feature. This study also identified the presence of surface Li^+^ ions as an important cytotoxicity-generating feature. Overall, these predictions indicated that the crucial factor for cytotoxicity of MXenes is not the structure of the materials, but the surface properties.

Interactions between MXenes and immune cells were also explored. Rafieerad et al. employed MXene (Ti_3_C_2_) QDs for immunomodulation and tissue repair [46]. In a simulated lymphocyte population, Ti_3_C_2_ QDs was shown to selectively reduce the activation of CD4^+^IFN-γ^+^ T-lymphocytes, while promoting the expansion of immunosuppressive regulatory T-cells. After being incorporated into a chitosan-based hydrogel, the constructed platform enhanced the stem cell delivery and tissue repair. The immunomodulatory properties of MXenes remind us to pay attention to the immunotoxicity of MXenes. Overall, the cytotoxicity of MXene has been found to depend on dose, material type, and cell type. At low doses, MXenes exhibits low cytotoxicity and good biocompatibility. Meanwhile, MXenes showed higher toxicity to cancer cells as compared to normal cells, and are more toxic to stem cells than to differentiated cells. Ultrasmall MXene QDs tend to exhibit higher cytotoxicity than nanosheet materials. Unlike other 2D nanomaterials, the cytotoxicity of MXenes is not strongly associated with the intracellular ROS, but instead with the stable binding of MXenes to cell membranes. Upon near-infrared (NIR) irradiation, certain MXenes showed strong photothermal therapy effects in promoting cancer cell ablation. Very recently, artificial intelligence methods were also used to verify and even to predict the cytotoxicity, and surface properties of MXene were identified as the crucial factors leading to cytotoxicity. Computer-based modeling thus represents a new way to gain additional knowledge regarding the in vitro toxicity of MXenes.

**Table 1:** Summary of in vitro toxicity of MXenes.

Type of MXenes	Type of Cells	Dose	Toxicity Effects	Reference
Ti_3_C_2_T_*x*_	human umbilical vein endothelial cells (HUVECs)	100 and 500 μg mL^−1^, 48 h	No obvious acute cytotoxicity. The ratios of living, apoptotic, and necrotic cells exhibited patterns similar to those of the control group.	[35]
Ti_3_C_2_ QDs, Nb_2_C QDs	HUVECs	0–100 μg mL^−1^, 24 h	At 25 μg mL^−1^ of Ti_3_C_2_ QDs, the cellular viability was larger than 70%. While at 50 and 100 μg mL^−1^, Ti_3_C_2_ QDs led to significantly lower cellular viability compared with Nb_2_C QDs. For Nb_2_C QDs, there was no significant cytotoxicity after treated with these three concentrations.	[36]
Ti_3_C_2_T_*x*_	human mesenchymal stem cells (hMSCs)	0–100 μg mL^−1^, 7 days	>50 μg mL^−1^, obvious cytotoxicity was shown	[37]
Ti_3_C_2_T_*x*_	neural stem cells (NSCs) and NSCs-derived differentiated cells	12.5–100 μg mL^−1^, 24 h	At 25 μg mL^−1^, Ti_3_C_2_T*_x_* nanosheets caused significant cytotoxicity to NSCs. 198 differently expressed genes (DEGs) which were mainly associated with the extracellular regions were identified.	[38]
Ti_3_C_2_T_*x*_	A549, MRC-5, A375, and HaCaT cells	0–500 μg mL^−1^, 24 h	Concentration dependent cytotoxicity. Toxic effects were higher against cancerous cells in comparison to normal ones.	[39]
Ti_3_C_2_T*_x_* MXene structures and their precursors (TiC, Ti_2_AlC, and Ti_3_AlC_2_)	HeLa cells and normal fibroblasts (MSU1.1)	10–400 μg mL^−1^, 24–48 h	Concentration dependent and cell-type dependent cytotoxicity. MXene structures showed higher cell viability in comparison to MAX phases.	[40]
Ti_2_NT*_x_*	human skin malignant melanoma cells, human immortalized keratinocytes, human breast cancer cells, and normal human mammary epithelial cells	62.5–500 μg mL^−1^, 24 h	Higher toxicity towards cancerous cell lines in comparison to normal ones.	[41]
Nb_2_CT*_x_*	breast 4T1 cancer cells and glioma U87 cancer cells	0–200 μg mL^−1^, 24 h	Negligible effect on the cell viability at 200 μg mL^−1^. After exposed to 808 or 1064 nm laser, cancer cells were killed significantly with the increase of laser intensity.	[13]
Ti_2_N QDs	293T, 4T1, and U87	0–80 μg mL^−1^, 24 h	Adding irradiating with 808 or 1064 nm lasers for 5 min after incubation with the Ti_2_N QDs, cancer cells were almost completely killed.	[43]
Ti_2_CT*_x_*	3D HeLa cell culture system	0–500 μg mL^−1^, 24 h	The direct adhesion of Ti_2_CT*_x_* MXenes to the cell membrane of microtissues limited the growth of microtissues and cell division, resulting in lower cell viability.	[44]

## 4. Toxic Effects of MXenes In Vivo

Zebrafish (*Danio rerio*) are the most often used model animals in in vivo investigations of the toxicity of nanomaterials. The utility of zebrafish in this regard is due to their high genetic homology to humans, small size, rapid reproduction and development and their transparency during early growth stages [47]. Nasrallah et al. employed a zebrafish embryo model to evaluate the potential in vivo acute toxicity of Ti_3_C_2_T*_x_* nanosheets [48]. No cumulative mortality was observed at a dose of 50 μg mL^−1^. At 100 μg mL^−1^, however, the cumulative mortality rate was 21%, and this dose represented the lowest concentration with an observed effect. According to a dose curve generated at 96 h post fertilization, the calculated dose that led to 50% lethality (LC_50_) was 257.46 μg mL^−1^. In addition to lethality, other assays revealed that Ti_3_C_2_T*_x_*-treated embryos exhibited a normal locomotion behavior at the lowest dose (50 μg mL^−1^), nor did Ti_3_C_2_T*_x_* nanosheets at this concentration influence neuron or muscle activity of the zebrafish embryos. Therefore, according to the Acute Toxicity Rating Scale by the Fish and Wildlife Service (FWS), Ti_3_C_2_T*_x_* nanosheets can be categorized as the “practically nontoxic” group.

Alhussain et al. employed chicken embryos to investigate the developmental toxicity of MXenes [49]. At a dose of 30 μg per embryo, after 5 days incubation, Ti_3_C_2_T*_x_* nanosheets provoked a significant toxicity on the chicken embryos, as approximately 46% of embryos died shortly after exposure. Moreover, at the tested dose, a significant inhibition of angiogenesis in the chorioallantoic membrane was also observed. These toxic effects may be attributed to the downregulation of several genes that control cell proliferation, survival, cell death and angiogenesis, including *ATF3*, *FOXA2*, *INHBA*, *SERPINA3* and *VEGF-C*. These findings demonstrated that MXenes potentially exert development toxicity on embryos. However, only one dose was tested in this study, and the method of exposure was debatable.

The recent reports of the use of MXenes for photothermal conversion and cancer photothermal therapy using near infrared radiation has led to a need for the in vivo evaluation of toxicity in a mouse model [13]. In Kunning mice, normal hematological parameters and standard blood biochemical indexes were confirmed and no statistical significance in these assays was observed in groups with 20 mg kg^−1^ Nb_2_CT*_x_* at 1, 7, and 28 days post-injection in comparison to the control group. These results indicated that no significant inflammation was caused by exposure to Nb_2_CT*_x_* nanosheets.

Hematoxylin and eosin staining assays were performed to evaluate histological changes of major organs, including the heart, liver, spleen, lung, and kidney. No significant histological abnormality was found during the treatment period after exposure to Nb_2_CT*_x_* nanosheets with or without near infrared light irradiation. These results revealed that MXenes are biocompatible and thus appropriate for in vivo photothermal ablation of cancer.

The biocompatibility of Ti_3_C_2_T*_x_* MXenes films was also evaluated when they were used for guided bone regeneration [50]. After implanting the films in subcutaneous sites and sites of calvarial defects in rats, Ti_3_C_2_T*_x_* MXene films was found to have no obvious inflammatory or toxic side effects, indicating their safety for in vivo uses.

**Figure 4:**
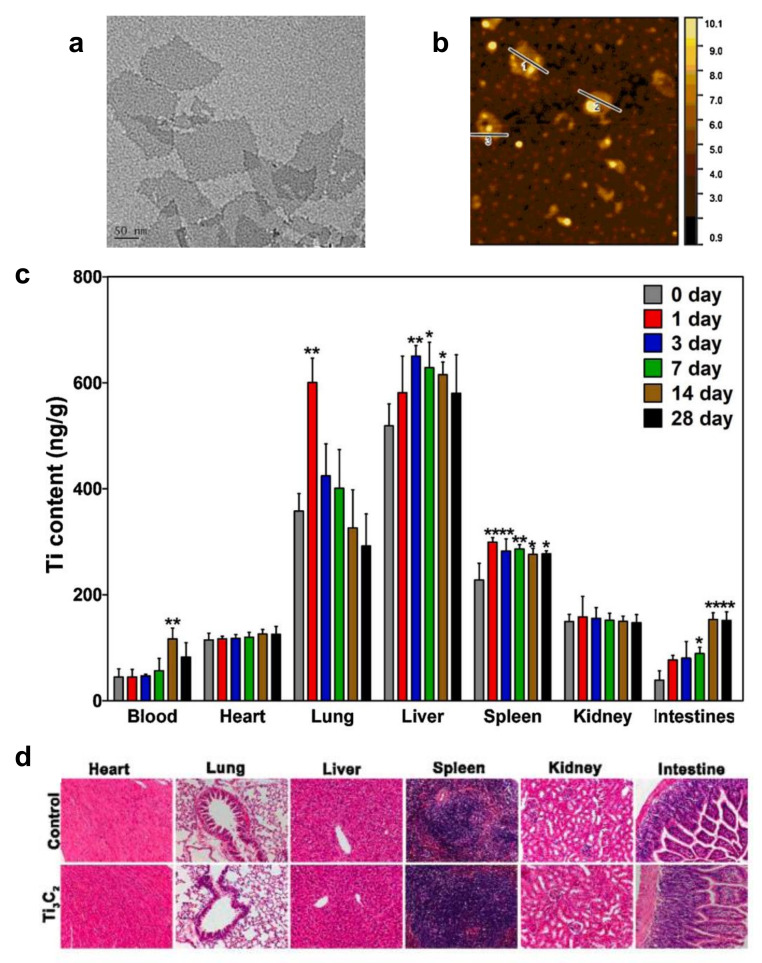
(**a**) TEM image of Ti_3_C_2_T*_x_* nanosheets. (**b**) AFM image of Ti_3_C_2_T*_x_* nanosheets. (**c**) Biodistribution of Ti_3_C_2_T*_x_* nanosheets in blood, heart, lung, liver, spleen, kidney, and intestines at 1, 3, 7, 14, 21 and 28 days after intravenous injection into mice. * *p* < 0.05, ** *p* < 0.01. (**d**) Representative sections from mice tissues after treatment of Ti_3_C_2_T*_x_* nanosheets. Reproduced with permission from [51]. Copyright Elsevier, 2021.

To evaluate the impacts of MXenes on organ function, Sui et al. investigated the distribution and accumulation of Ti_3_C_2_T*_x_* nanosheets in major organs [51]. ICR mice were exposed to Ti_3_C_2_T*_x_* nanosheets by intravenous injection at a dose of 20 mg kg^−1^. At 1, 3, 7, 14 and 28 days, the blood, lungs, heart, kidneys, liver, spleen and intestines were harvested, and Ti contents were measured. The researchers found that Ti_3_C_2_T*_x_* nanosheets were primarily distributed in the lung and liver (Figure 4). Those in the lungs were internalized by endothelial and alveolar epithelial cells, resulting in the decrease of surfactant protein B content in alveolar epithelial cells and causing an increase in Penh, an indicator of lung dysfunction. A decrease of respiratory rate was also observed. These results indicated that when Ti_3_C_2_T*_x_* nanosheets crossed the lung, they potentially exerted toxicity that led to respiratory disorders. Ti_3_C_2_T*_x_* nanosheets that accumulated in liver were endocytosed by sinusoidal endothelial cells and were gradually excreted via bile canaliculi. During the entire exposure period, inflammatory responses and pathological lesions, including within the small and large airways of the lung, were observed as not obviously abnormal. Several issues with the experimental design in this case mean that results should be interpreted with caution. For example, the stability of the material was not investigated. In addition, while the diameter of the Ti_3_C_2_T*_x_* nanosheets was stated to 100 to 200 nm, no statistical analysis of the tunneling electron microscope images of the nanosheets was presented.

As environmental release of MXenes or byproducts of synthesis is inevitable, toxicity studies in plants are also important. MXenes are fabricated by etching the A-layer of MAX phase, and MAX phase materials are wildly used in the fields of ceramics, catalysis, environment remediation, and biomedicine. Therefore, seed germination and growth of rice were employed to assess the potential phytotoxicity of MAX phase materials [52]. After foliar spraying at doses of 100 and 1000 μg·mL^−1^, Ti_3_AlC_2_ nanosheets inhibited the growth of seedlings. In contrast to previous studies of toxicity of MXenes, the inhibition in this case was found to be due to the generation of ROS. At a dose of 100 μg·mL^−1^, the stomatal aperture was increased to 78.6%. Meanwhile, the number of trichomes was increased by 100%. These responses were mainly caused by the entrance of titanium ions released from the Ti_3_AlC_2_ nanosheets. To reduce the entrance of external ions, rice changed the structure and function of the leaves so as to activate the immune system and enhance the activity of antioxidant enzymes. However, at high doses, the plant was not able resist abiotic stress, the cell membranes were damaged, and biomass and chlorophyll content were decreased. These eventually led the rice growth inhibition.

There are few studies on the in vivo toxicity of MXenes. The iv vivo toxicity evaluation of MXenes mentioned above was summarized in Table 2. In particular, barely any studies use environmental doses and environmentally relevant modes of exposure to evaluate the consequences of long-term exposure. Although in vivo toxicity can be observed at high doses in some studies, these assays do not provide enough evidence to suggest the safety of MXenes in biomedical applications.

## 5. Properties Associated with the Safety of MXenes

### 5.1. Surface Functionalization

To improve the biocompatibility of MXenes, collagen has been conjugated to the surfaces of Ti_3_C_2_T*_x_* and Ti_2_CT*_x_* nanosheets [53]. The interactions between the MXenes and collagen mainly involved electrostatic forces, and zeta potential measurements were used to characterize and control the adsorption and desorption of collagen. The viability of A375 (human skin malignant melanoma cells), HaCaT (human immortalized keratinocytes), MCF-7 (human breast cancer cells) and MCF-10A (mammary epithelial cells) were evaluated upon treatment with surface-modified and unmodified nanosheets. It was found that the surface collagen modification significantly decreased the cytotoxicity, as well as reduced oxidative stress. The mechanism of increasing the biocompatibility of MXenes by collagen modification should be further investigated.

Rashid et al. conjugated polypropylene glycol (PPG) and polyethylene glycol (PEG) to the surfaces of Ti_3_C_2_T*_x_* nanosheets [54] and assessed their cytotoxicity towards normal (HaCaT and MCF-10A) and cancerous (MCF-7 and A375) cells. PEG-modified Ti_3_C_2_T*_x_* nanosheets were the most toxic to normal and cancer cell lines, followed by PPG-modified nanosheets, and the least toxic were bare MXenes. As photothermal therapy agents, PPG-modified nanosheets were safer to normal cells as compared to PEG-modified nanosheets. As polymer coating typically reduces the toxicity of nanomaterials, conclusions from this study should be further evaluated with additional in vitro or in vivo assays.

To integrate the photothermal therapy capabilities of MXenes with targeting and imaging functions, Hussein et al. fabricated two plasmonic Ti_3_C_2_T*_x_*-based nanocomposites, i.e., Au/MXene and Au/Fe_3_O_4_/MXene nanocomposites [55]. In a human breast cancer cell lines MCF7, these nanocomposites showed similar photothermal therapy efficiencies. However, hybrid nanocomposites showed less in vivo toxicity than did pure MXene. The in vivo acute toxicity assays were conducted using zebrafish embryos. Au/MXene and Au/Fe_3_O_4_/MXene led to less embryonic mortality with LC_50_ values greater than 1000 μg mL^−1^ than pure MXene, with an LC_50_ value of 257.46 μg mL^−1^. No explanations for this phenomenon were proposed and the mechanisms should be further explored.

In addition, Ti_2_CT*_x_* nanosheets can be surface modified by post-delamination using mild heating at 60 °C [56]. After thermal treatment, the cytotoxicity of Ti_2_CT*_x_* nanosheets showed selectively toxic to cancer cells compared to normal cells. At a concentration of 62.5 μg mL^−1^, post-treatments, Ti_2_CT*_x_* nanosheets were lethal to A375 cells, but not to normal HaCaT cells. This difference was because of a Ti_2_O_3_ layer that was formed on the Ti_2_CT*_x_* nanosheets after mild oxidation at 60 °C. The presence of the Ti_2_O_3_ layer caused higher generation of ROS as compared to non-oxide Ti_2_CT*_x_* nanosheets.

### 5.2. Stability and Degradation

**Figure 5:**
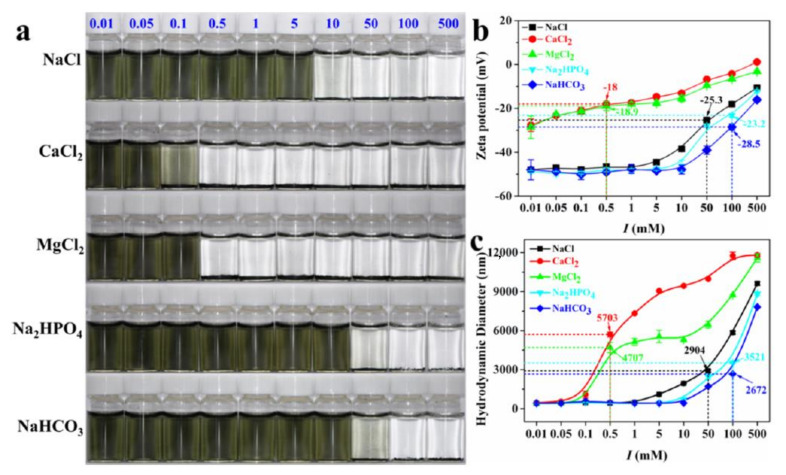
(**a**) Photo illustration of the effects of cations and anions with different concentrations (mM) on the aggregation behaviors of Ti_3_C_2_T*_x_* nanosheets. (**b**) Zeta potential of Ti_3_C_2_T*_x_* nanosheets in the presence of different electrolytes. (**c**) Hydrodynamic diameter of Ti_3_C_2_T*_x_* nanosheets in the presence of different electrolytes. Reproduced with permission from [58]. Copyright American Chemical Society, 2020.

The dispersion state and sizes of the aggregates are important factors that influence cellular uptake and subsequent cytotoxicity [57]. It is thus important to evaluate aggregation states before performing toxicology evaluations. Nasrallah et al. tested the stability of Ti_3_C_2_T*_x_* nanosheets in seawater and E3 medium by monitoring the scattering signal of nanosheet aggregates [48]. It was observed that, at 50 μg mL^−1^, a suspension of Ti_3_C_2_T*_x_* nanosheets with diameters of 200 nm was more stable than at higher concentrations. There was no significant change in the scattering signal over the 18 h incubation both in seawater and E3 medium. Xie et al. systematically investigated the colloidal properties and stability of Ti_3_C_2_T*_x_* nanosheets in aqueous solutions by adjusting the pH, ionic types, and ionic strength [58]. It was found the stability of Ti_3_C_2_T*_x_* nanosheets varies with ionic types and ionic strength after a 24 h incubation in different electrolytes (Figure 5). The effects of the electrolytes in destabilizing Ti_3_C_2_T*_x_* nanosheets follows the order of CaCl_2_ > MgCl_2_ > NaCl > Na_2_HPO_4_ > NaHCO_3_. When the electrolyte reached a critical concentration, Ti_3_C_2_T*_x_* nanosheet suspensions became unstable and formed visible agglomerates. The aggregation states were accompanied by an increasing of zeta potentials and hydrodynamic diameters. Since the MXenes were suspended in culture medium for longer than 24 h, it would have been important to evaluate the stability of the MXene suspensions in the culture medium.

MXenes are susceptible to environmental oxidation and can be decomposed to transition metal oxides and carbide-derived carbon. The decomposed byproducts can also induce toxic effects in vitro and in vivo. Jastrzebska et al. studied the oxidation of the most unstable V_2_CT*_x_* nanosheets in cell culture media and the resulting toxicity [59]. V_2_CT*_x_* nanosheets in cell culture medium were found to undergo a rapid oxidation, leading to decomposition and entry into the mixture of vanadium oxides and carbide-derived-carbon after 48 h. The formed vanadium oxides were highly cytotoxic and the decreasing of cell viability was oxidation dependent. The mechanisms of toxicity were related to the disruption of cell cycle and cell membrane disintegration through contact with oxidized flakes.

In addition, biodegradation of MXenes was also investigated by incubation of Nb_2_CT*_x_* nanosheets with human myeloperoxidase (hMPO) and H_2_O_2_ in PBS [13]. The suspension turned translucent in 24 h, indicating the degradation of Nb_2_CT*_x_* nanosheets. However, the decomposed products and their toxicity were not studied in this report.

## 6. Conclusions and Future Perspectives

Understanding the fundamental interactions between MXenes and biological systems and the toxic effects of MXenes in vitro and in vivo are of utmost importance for further development of MXene-based nanomedicines. These insights are also required for the development of safety guidelines for researchers working with this new class of 2D nanomaterial. Like other 2D nanomaterials, MXenes can enter cells and induce cytotoxicity. The available in vivo data suggests that MXenes exhibit potential pulmonary, reproductive, and developmental toxicities in laboratory animal and plant models. However, the information about the toxicity of MXenes remains very limited, especially when taking into account the many different types of MXenes and their potential modifications. The composition, size, surface chemistry, dose, stability, cell types, and means of exposure are all factors determining the in vitro and in vivo toxicity of MXenes. Oxidative stress and cell membrane disruption may be involved in the mechanisms of the toxicity of MXenes.

Toxicology evaluation methods have been well-established. However, we should pay particular attention to material characterization before using these methods, as toxicity varies with the physiochemical properties of MXenes. Key parameters, including size, morphology, layer number, zeta potential, surface chemistry, and dispersion states, influence the interactions between MXenes and biological systems and can lead to different toxicities. Many studies cited in this review did not provide full information regarding the physiochemical properties of the MXenes that were tested. In addition, while protein corona formation is known as a key factor affecting the interactions between MXenes and biological systems [60,61,62], no studies have considered this factor in toxicology studies. The influence of protein corona composition on MXenes and their behaviors in biological systems needs to be clarified so as to further the understanding of toxicological mechanisms.

This review demonstrated that studies have tended to focus on acute toxicological effects, but no long-term toxicology investigations at environmental doses have been performed. In addition to intravenous injection, oral and respiratory routes of exposure should also be considered. To completely understand the toxicity of MXenes, studies should be conducted using various types of well-characterized MXenes. In addition, toxicities to dermal, ocular, genomic and immunology systems should be performed. Considering the large number of MXene types and derivatives and the time consuming and expensive nature of toxicology evaluations, computer-aided toxicology methods, especially artificial intelligence models, should be employed as powerful tools for toxicology prediction and mechanism discovery [63,64]. Furthermore, the in vivo degradation, metabolization and excretion behaviors of MXenens also need to be established in future research. In short, more systematic investigations are still in high demand in order to address safety concerns before the wide practical application of MXenes.

## Figures and Tables

**Table 2:** Summary of the in vivo toxicity of MXenes.

Type of MXenes	Type of Models	Dose	Toxicity Effects	Reference
Ti_3_C_2_T*_x_*	Zebrafish embryos	25–200 μg mL^−1^	The calculated LC_50_ was 257.46 μg mL^−1^. At the concentration of 50 μg mL^−1^, no acutoxicity or neurotoxicity was observed.	[48]
Ti_3_C_2_T*_x_*	Chicken embryos	30 μg per embryo, 5 days incubation	Potential toxicity on the early stage of embryogenesis; down regulation of several controller genes of cell proliferation, survival, cell death and angiogenesis; inhibition of blood vessel development.	[49]
Nb_2_CT*_x_*	Kunming mice	20 mg kg^−1^	No significant inflammation was caused. No significant histological abnormality was found. Nb_2_CT*_x_* are highly biocompatible.	[13]
Ti_3_C_2_T*_x_*	ICR mice	20 mg kg^−1^	Ti_3_C_2_T*_x_* nanosheets could accumulate in the liver lungs. Those in the lung might influence respiratory function via the downregulation of surfactant protein B in alveolar epithelial cells.	[51]
Ti_3_AlC_2_	Rice	0.1–1000 μg mL^−1^	At the doses of 100 and 1000 μg·mL^−1^, Ti_3_AlC_2_ nanosheets inhibited the growth of seedlings due to the generation of ROS. At the dose of 100 μg·mL^−1^, the stomatal aperture was increased to 78.6%. Meanwhile, the number of trichomes was increased to 100%.	[52]
